# The Double Life of microRNAs in Bone Sarcomas: Oncogenic Drivers and Tumor Suppressors

**DOI:** 10.3390/ijms26104814

**Published:** 2025-05-17

**Authors:** Stefano Zoroddu, Fabio Sias, Luigi Bagella

**Affiliations:** 1 Department of Biomedical Sciences, University of Sassari, Viale San Pietro 43/b, 07100 Sassari, Italy; 2 Sbarro Institute for Cancer Research and Molecular Medicine, Centre for Biotechnology, College of Science and Technology, Temple University, Philadelphia, PA 19122, USA

**Keywords:** bone sarcomas, osteosarcoma, chondrosarcoma, Ewing sarcoma, microRNA, novel therapies, cancer, molecular oncology, cancer biomarkers, microRNA dysregulation

## Abstract

Bone sarcomas, including Osteosarcoma, Ewing’s sarcoma, and Chondrosarcoma, are rare yet aggressive tumors with high metastatic potential and poor survival outcomes. Despite advances in surgical and chemotherapeutic techniques, these malignancies remain difficult to treat. They often exhibit resistance to conventional therapies and are associated with a limited prognosis for patients. MicroRNAs (miRNAs) have emerged as pivotal regulators of cancer biology, orchestrating crucial processes such as cell proliferation, apoptosis, and metastasis. Their double life as oncogenes or tumor suppressors underscores their significance in the pathogenesis of bone sarcomas. This review examines the multifaceted roles of miRNAs in these malignancies. By elucidating the complex networks affected by miRNA dysregulation, we seek to identify novel avenues for miRNA-based interventions. It is the intention of this work to stimulate future research and clinical strategies that exploit the potential of miRNAs to transform the management and outcomes of bone sarcomas.

## 1. Introduction

Bone sarcomas represent a rare but highly aggressive form of primary bone cancer, with a predilection for children, adolescents, and young adults [1,2]. Among the most clinically relevant bone sarcomas are Osteosarcoma, Ewing’s sarcoma, and Chondrosarcoma, each of which exhibits distinct pathological, clinical, and molecular characteristics [3,4,5]. Despite recent advances in diagnostic techniques and therapeutic modalities, these neoplasms frequently have a poor prognosis due to high rates of metastasis, recurrence, and resistance to treatment. Osteosarcoma is the most prevalent form of the disease (Table 1) and typically affects adolescents during periods of rapid bone growth [6]. It is distinguished by the formation of immature osteoid. The implementation of multimodal treatments, which involve surgical intervention, chemotherapy, and radiotherapy, has resulted in an improvement in patient outcomes. Nevertheless, the survival rate for individuals with metastatic or recurrent cases remains low, at approximately 20%, due to the emergence of chemoresistance and the robust metastatic capacity of these cancers [7,8]. Several studies have highlighted how molecular heterogeneity in bone sarcomas, particularly the involvement of epigenetic regulators and chromatin-modifying complexes, may influence therapeutic responses and disease progression [9].

**Figure 1:**
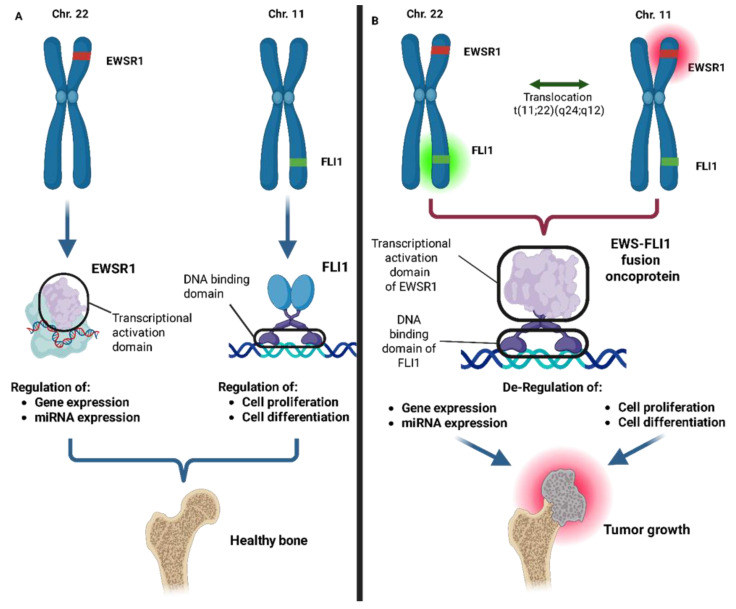
(**A**) Under physiological conditions, the genes encoding the EWS and FLI1 proteins are located on chromosomes 22 and 11, respectively, where they regulate normal cellular functions. (**B**) A translocation between chromosomes 22 and 11. This leads to the combination of the transcriptional activation domain of EWSR1 with DNA-binding domain of FLI1 resulting in the formation of the EWS-FLI1 fusion oncoprotein which loses its regulatory function and disrupts cellular processes, leading to the development of Ewing’s sarcoma.

Ewing’s sarcoma, the second most prevalent bone tumor in children and young adults, is driven by chromosomal translocations that produce abnormal fusion proteins, such as EWS-FLI1, which substantially alter carcinogenesis and miRNA expression (Figure 1) [10,11]. Recent data suggest that epigenetic and transcriptional vulnerabilities can be exploited for therapeutic purposes, especially in tumors with MYC overexpression or fusional-driven gene dysregulation [12]. Notwithstanding the improved survival rates for localized disease resulting from advances in chemoradiotherapy and surgical techniques, the outcomes for metastatic or relapsed cases remain poor [13,14,15].

Chondrosarcoma is defined by the formation of cartilaginous matrix. It differs from Osteosarcoma and Ewing’s sarcoma because it primarily affects adults and it is resistant to chemotherapy and radiotherapy, which makes surgical intervention the primary means of treatment [16,17,18]. The prognosis varies considerably in accordance with the grade of the tumor. High-grade forms are aggressive and prone to recurrence and metastasis, and the range of available therapeutic options is limited.

**Figure 2:**
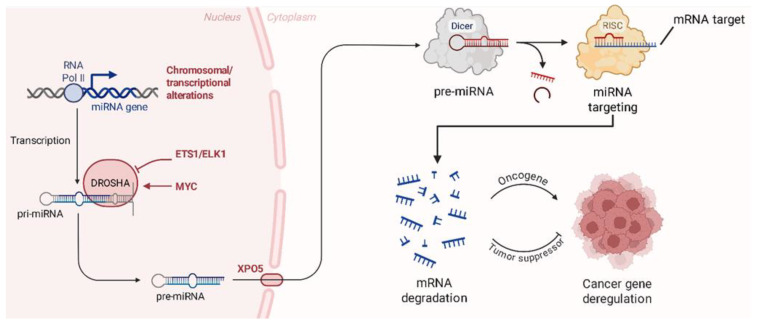
Biogenesis and mechanism of action of miRNAs in cancer. miRNAs are transcribed from miRNA genes by RNA polymerase II, producing primary miRNAs (pri-miRNAs) that are processed in the nucleus by the DROSHA complex into precursor miRNAs (pre-miRNAs). After nuclear export via Exportin-5 (XPO5), pre-miRNAs are further cleaved by Dicer in the cytoplasm, generating mature miRNAs. These are incorporated into the RNA-induced silencing complex (RISC), guiding it to partially complementary sequences within the 3′ untranslated region (3′ UTR) of target mRNAs. This interaction results in mRNA degradation or translational repression. Alterations in miRNA expression or processing can lead to the dysregulation of oncogenes or tumor suppressor genes, thereby contributing to cancer development.

The treatment of these sarcomas often requires a mix of strong surgical resection and adjuvant chemotherapy or radiotherapy, with the exact histological subtype and stage of the tumor being considered [19]. However, systemic toxicity, tumor heterogeneity, and medication resistance have a considerable impact on the outcome of therapeutic efforts. The creation of trustworthy biomarkers for early identification, prognosis, and medication response is of essential relevance to enhance patients’ outcomes [20]. MicroRNAs (miRNAs) have been found to be pivotal regulators of gene expression, influencing several critical biological processes, including cell proliferation, death, invasion, and metastasis [21]. The dysregulation of miRNAs has been discovered in all these three bone sarcoma subtypes and in several soft tissue sarcomas [22]. These miRNAs can behave as either oncogenes, which promote tumor growth and therapy resistance, or tumor suppressors, which decrease malignancy and sensitize tumors to treatment [22,23]. In addition, recent transcriptomic and pharmacological analyses have shown that small molecules, such as oxadiazole analogs, can modulate mitotic pathways and influence tumor cell plasticity, further underscoring the importance of regulatory RNAs and cytoskeletal dynamics in tumor biology [24,25]. Based on this, related compounds such as benzotriazole–acrylonitrile derivatives have also demonstrated the ability to interfere with microtubule function by simultaneously inducing extensive transcriptomic changes, including pathways commonly influenced by miRNAs [26]. Moreover, miRNAs represent promising therapeutic tools due to their ability to finely regulate gene expression post-transcriptionally. As illustrated in Figure 2, miRNAs are generated through a multistep biogenesis process and exert their function by binding the 3′ untranslated regions (3′ UTRs) of target mRNAs, leading to either mRNA degradation or translational repression. Therapeutic strategies such as miRNA mimics or inhibitors aim to restore normal miRNA function, thereby correcting the dysregulation of oncogenes or tumor suppressor genes commonly observed in cancers, including bone sarcomas [27].

This review aims to synthesize existing knowledge on the dysregulation of miRNAs in bone sarcomas, exploring their functional roles in tumor biology and their potential as diagnostic, prognostic, and therapeutic targets. Each miRNA involved in these malignancies will be analyzed, providing insights into their specific contributions to tumorigenesis and their impact on clinical outcomes. By highlighting miRNA-mediated regulatory networks, this work seeks to open novel avenues for early diagnosis, better prognostication, and the development of targeted therapies. Given their double life as oncogenes and tumor suppressors, miRNAs represent a promising frontier in the fight against these challenging malignancies.

## 2. miRNAs Dysregulation in Bone Sarcomas

In bone sarcomas, miRNAs play a pivotal role in modulating tumor behavior by influencing key biological processes such as cell proliferation, differentiation, apoptosis, and metastasis. Research has uncovered a range of miRNAs with aberrant expression patterns in different types of bone sarcomas, such as Osteosarcoma, Ewing’s sarcoma and Chondrosarcoma. These miRNAs can function as either oncogenes or tumor suppressors, depending on their targets and the context of their expression, contributing significantly to the pathogenesis and progression of these malignancies (Table 2). Notably, several miRNAs regulate overlapping signaling pathways, such as PI3K/AKT, p53-mediated apoptosis, and Wnt/β-catenin [28,29]. This convergence suggests potential synergistic or antagonistic effects between miRNAs that modulate tumor progression, chemoresistance, or metastasis. For instance, multiple tumor-suppressive miRNAs target anti-apoptotic factors like BCL-2, while distinct oncogenic miRNAs may enhance cell survival through parallel routes [30]. Understanding these cooperative or competitive interactions may be critical for designing combinatorial miRNA-based therapies.

### 2.1. miRNAs in Osteosarcoma

Osteosarcoma is the most prevalent primary malignant bone tumor, primarily affecting teenagers and young adults [31,32]. Characterized by high aggressiveness, fast development, and a predisposition for early metastasis, this malignancy provides major hurdles in both diagnosis and treatment [33]. Despite breakthroughs in chemotherapy and surgical procedures, patient outcomes remain unsatisfactory, particularly for individuals with metastatic or recurring cancer. The molecular intricacy of Osteosarcoma underlines the need for greater knowledge into its underlying biology. In this context, we investigate the roles of the most prominent miRNAs in Osteosarcoma, outlining their contributions to tumor pathogenesis and evaluating their possible applications in clinical therapy.

### 2.2. Oncogenic miRNAs in Osteosarcoma

**Figure 3:**
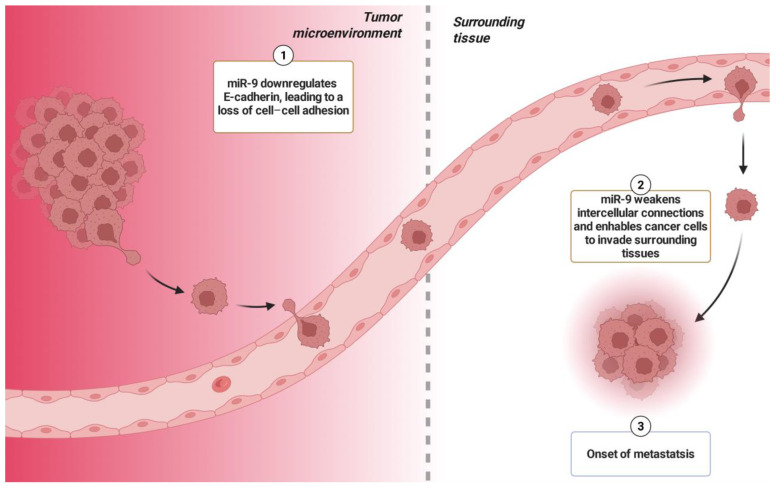
miR-9 enhances cancer cell proliferation and metastasis by targeting key molecules involved in cell–cell adhesion. Specifically, miR-9 downregulates E-cadherin expression, weakening intercellular connections and facilitating tumor cell invasion into surrounding tissues. This dual role in promoting both proliferation and metastatic potential underscores miR-9 as a critical regulator in cancer progression.

Several miRNAs have been identified as oncogenic in Osteosarcoma, promoting tumor progression and metastasis:

**miR-9** has attracted significant attention as a key oncogene in Osteosarcoma, playing a pivotal role in orchestrating numerous pathways that accelerate tumor development and dissemination. An enhanced expression of miR-9 has been found in Osteosarcoma tissues and is significantly related to unfavorable prognostic outcomes [34]. Its activity carefully governs a network of molecular interactions that impair cellular homeostasis and contribute to cancer development. The main method by which miR-9 aids carcinogenesis is by its ability to regulate the cell cycle, hence facilitating unregulated proliferation [35]. By boosting the shift from the G1 phase, miR-9 successfully overcomes the regulatory systems that generally control aberrant cell proliferation [36]. Furthermore, its effects on proliferation are magnified by its role in metastasis, as miR-9 targets key molecules such as E-cadherin, an essential component of cell–cell adhesion. miR-9 downregulates E-cadherin expression, thereby weakening intercellular connections and enabling cancer cells to invade surrounding tissues (Figure 3). This mechanism facilitates the epithelial–mesenchymal transition (EMT), a critical pathway for the invasive capabilities of cancer cells [37].

While miR-9’s regulation of MMP-13 (matrix metalloproteinase-13) remains less well-documented in Osteosarcoma, its influence on other metalloproteinases, such as MMP-2 and MMP-9, underscores its capacity to degrade the extracellular matrix and promote invasion [38]. Evidence from other malignancies suggests miR-9’s involvement in similar extracellular matrix remodeling processes, supporting its broader role in tumor progression. In Osteosarcoma, miR-9 has been shown to modulate the Wnt signaling pathway, a driver of carcinogenesis, proliferation, and migration [39]. Xu et al. emphasized miR-9’s enhancement of Wnt signaling, leading to increased Osteosarcoma cell survival and metastatic behavior [34]. Furthermore, miR-9 contributes to therapy resistance by regulating autophagy-related pathways [39]. Clinically, miR-9 overexpression is associated with worse survival rates in Osteosarcoma patients, marking it as a potential prognostic biomarker. Its dual role as a driver of metastasis and resistance to therapy identifies miR-9 as a compelling target for therapeutic intervention [34]. Although direct evidence for the use of antagonists targeting miR-9 in Osteosarcoma is currently lacking, the successful application of antagonists against other oncogenic miRNAs, such as miR-17-5p in Neuroblastoma models, suggests a promising avenue for similar strategies [40].

**miR-95** has emerged as a prominent oncogene in Osteosarcoma, playing a vital role in driving tumor formation and progression [41]. Its overexpression is related to quick proliferation, reduced apoptosis, and an aggressive clinical phenotype. By targeting the sodium channel epithelial 1α subunit (SCNN1A) and cyclin D1, miR-95 triggers the G1 phase transition, promoting uncontrolled cell proliferation [42]. Cyclin D1 overexpression induces fast growth of Osteosarcoma cells. Elevated levels in tumor tissues and blood samples are associated with advanced disease stages and poor prognosis [41].

**Figure 4:**
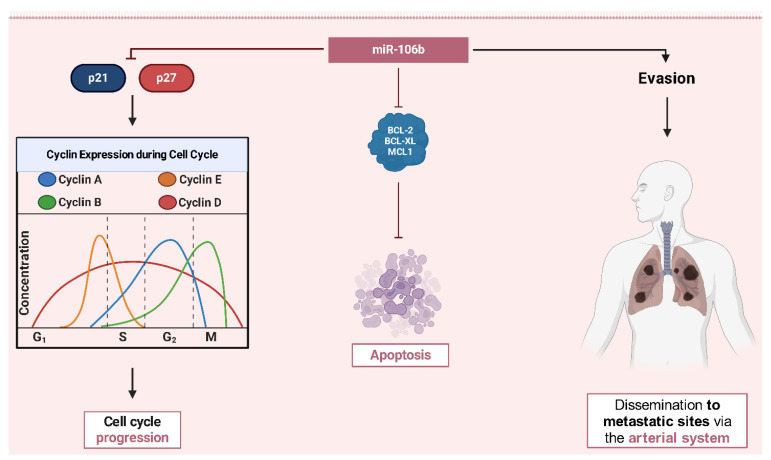
miR-106b serves as a critical regulator of tumor growth and metastasis in Osteosarcoma by modulating key cell cycle checkpoints and apoptotic pathways. By targeting cyclin-dependent kinase inhibitors such as p21 and p27, miR-106b facilitates the G1/S transition, leading to uncontrolled cell proliferation. Additionally, its role in promoting invasive behavior is linked to advanced disease stages, including extensive lung metastases. miR-106b also contributes to chemotherapy resistance by altering apoptotic signaling, particularly through the regulation of BCL-2 family proteins, thereby preventing programmed cell death.

**miR-106b** acts as the crossroads of tumor growth and metastasis, exerting its effects via changing crucial cell cycle checkpoints and promoting invasive tendencies [43]. Specifically, miR-106b targets cyclin-dependent kinase inhibitors like p21 and p27, resulting in the lowering of constraints on the G1/S transition [44]. This culminates in uncontrolled DNA replication and increased tumor cell division. Elevated expression of miR-106b is strongly connected with advanced Osteosarcoma, including instances with extensive lung metastases, suggesting its function in aggressive disease characteristics. Experimental research has shown that reducing miR-106b in Osteosarcoma cells affects both growth and metastatic potential, suggesting its dual role [27]. Additionally, miR-106b contributes to chemotherapy resistance by changing apoptotic pathways, such as those involving BCL-2 family proteins, which prevent programmed cell death (Figure 4) [45,46].

**Figure 5:**
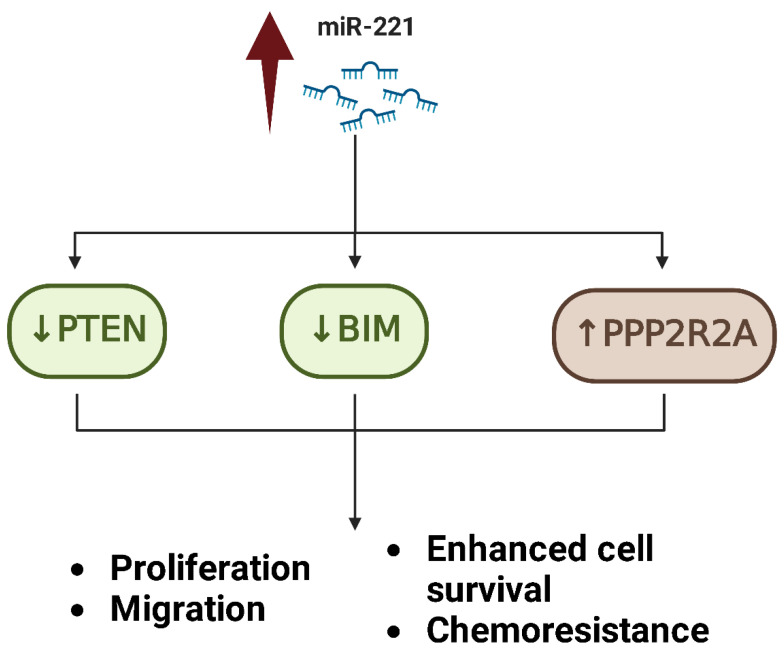
miR-221 promotes tumor migration, survival, and chemoresistance by targeting key tumor suppressor genes. Specifically, miR-221 downregulates PTEN and pro-apoptotic factors such as BIM and upregulates anti-apoptotic factors like PP2R2A, shifting the balance toward cell survival even under chemotherapeutic stress.

**miR-221** has been demonstrated to increase tumor invasion, survival, and chemoresistance by targeting numerous tumor suppressor genes [47,48]. One of its key targets is PTEN (phosphatase and tensin homolog), a vital negative regulator of the PI3K/AKT signaling pathway. By downregulating PTEN, miR-221 increases AKT hyperactivation, which results in enhanced cell survival, proliferation, and migration [47]. Additionally, miR-221 alters apoptotic pathways by targeting genes involved in apoptosis, including BIM and PPP2R2A, and therefore altering the balance in favor of cell survival, even under situations of chemotherapeutic stress (Figure 5). Elevated levels of miR-221 are related to severe cases of Osteosarcoma and widespread metastasis, particularly to the lungs [47]. Experimental evidence suggests that the lowering of miR-221 can restore chemosensitivity and greatly decrease tumor cell motility and invasion, identifying it an interesting therapeutic target [49,50].

**miR-299-5p** increases cell cycle progression by preferentially targeting cyclin-dependent kinase (CDK) inhibitors, such as p16 and p21, hence undermining the regulatory mechanisms that prohibit excessive proliferation [51]. This unregulated transition from the G1 to the S phase allows for fast DNA replication and tumor development. The overexpression of miR-299-5p correlates with advanced tumor stages and poor prognosis [51]. While its direct impact in chemoresistance demands further exploration, preliminary evidence shows that its influence on DNA replication pathways may give an innate survival advantage during chemotherapy. Silencing miR-299-5p in Osteosarcoma cells resulted in cell cycle arrest in the G1 phase, suggesting its potential as a therapeutic target [51].

**miR-4295** promotes proliferation and invasion by suppressing tumor suppressor genes such as IRF1, which play essential roles in regulating apoptosis and immune responses [52,53]. A study by Cheng et al. demonstrated that miR-4295 is upregulated in Osteosarcoma tissues and cell lines, leading to decreased expression of IRF1. This downregulation of IRF1 contributes to enhanced cell proliferation, migration, and invasion in Osteosarcoma cells.

Finally, **miR-181a** is another miRNA identified as playing an oncogenic role in Osteosarcoma; it plays a critical role in tumor progression by promoting proliferation, invasion, and resistance to apoptosis [54]. One of its main mechanisms involves targeting PTEN, a tumor suppressor that negatively regulates the PI3K/AKT signaling pathway. Suppression of PTEN by miR-181a leads to activation of AKT, increasing cell survival, proliferation, and migration [55]. Zhu et al. showed that the overexpression of miR-181a correlates with the increased phosphorylation of AKT, which promotes tumor growth and metastasis [54].

### 2.3. Tumor Suppressor miRNAs in Osteosarcoma

**miR-34a** acts as a potent tumor suppressor in Osteosarcoma, with its lower expression correlating closely with increased tumor aggressiveness, chemoresistance, and poor prognosis. Its tumor-suppressive activities are mediated by its capacity to target important genes and pathways involved in cell proliferation, apoptosis, and invasion [56,57,58]. A crucial discovery by Li et al. established that miR-34a targets TGIF2 (TGFB-induced factor homeobox 2), a transcriptional repressor implicated in TGF-β signaling [57]. TGIF2 is related to tumor growth, primarily through the stimulation of EMT, which promotes cell motility and metastasis. miR-34a’s inhibition of TGIF2 was demonstrated to impede these mechanisms, thereby lowering tumor invasion and metastatic potential in Osteosarcoma cells. In another key work, Yan et al. identified c-Met as a direct target of miR-34a. c-Met, a receptor tyrosine kinase activated by hepatocyte growth factor (HGF), plays a vital role in cell migration and survival [58]. Their results indicated that reinstalling miR-34a into Osteosarcoma cells led to a substantial drop in c-Met expression, leading in decreased migratory and invasive capabilities. This research underscores miR-34a’s function in attenuating the metastatic behavior of Osteosarcoma. miR-34a also regulates apoptosis by targeting BCL-2, a well-known anti-apoptotic protein [59]. Li et al. revealed that the downregulation of BCL-2 by miR-34a restores the apoptotic pathways in Osteosarcoma cells, resulting in enhanced cell death, particularly when paired with chemotherapeutic drugs like cisplatin [60]. This combined effect of increasing apoptosis and sensitizing cells to chemotherapy underscores miR-34a’s therapeutic potential. In addition to its impact on apoptosis, miR-34a modulates cell cycle progression by targeting cyclin D1, a critical regulator of the G1/S transition [56]. Wu et al. demonstrated that the overexpression of miR-34a in Osteosarcoma cells induces G1 phase arrest and significantly reduces proliferation by targeting Eag1 (Ether-à-go-go 1), a potassium channel implicated in cell cycle regulation [61]. The suppression of Eag1 by miR-34a leads to decreased cyclin D1 expression and inhibition of CDK4/6 activity, thereby impairing the G1/S transition. This molecular mechanism highlights miR-34a’s crucial role in restricting Osteosarcoma progression and maintaining cell cycle control [61].

**miR-335** has emerged as a critical tumor suppressor in Osteosarcoma, exerting its effects by regulating key signaling pathways involved in tumor growth, invasion, and metastasis [62,63]. One of its primary targets is ROCK1 (Rho-associated coiled-coil containing protein kinase 1), a pivotal player in cytoskeletal remodeling, cell motility, and metastatic progression [64,65].

In Osteosarcoma, ROCK1 is frequently upregulated, contributing to increased tumor cell migration, invasion and EMT [64,65]. By directly targeting ROCK1 mRNA, miR-335 acts as a negative regulator of these oncogenic processes. The downregulation of miR-335 in Osteosarcoma cells leads to unchecked ROCK1 expression, enhancing tumor aggressiveness and metastatic potential, particularly to the lungs [65]. Conversely, the restoration of miR-335 expression has been shown to significantly suppress Osteosarcoma cell invasion and metastasis by inhibiting ROCK1-mediated cytoskeletal rearrangements and cell motility [64].

The growing body of research on miR-335 underscores its significance as not only a key molecular regulator but also a potential biomarker for disease progression and treatment response. Further investigation into miR-335-based therapies may open new avenues for combating Osteosarcoma, particularly in patients with high metastatic risk.

**Figure 6:**
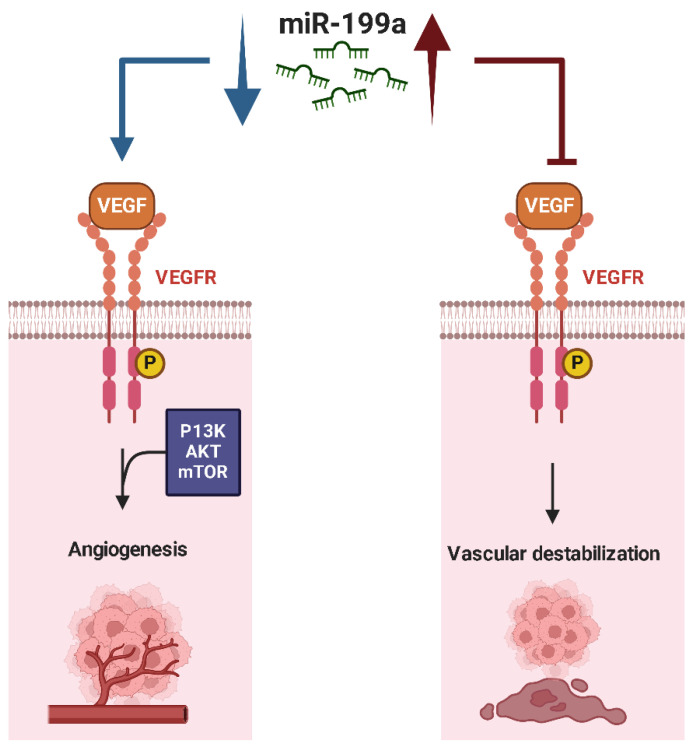
**Left:** miR-199a downregulation leads to uncontrolled VEGFR synthesis, triggering a strong angiogenic response that supports tumor progression and metastasis. **Right:** miR-199a upregulation inhibits VEGFR synthesis, resulting in the collapse of the capillary network that sustains the tumor.

In Osteosarcoma, **miR-199a** functions predominantly as a tumor suppressor. Its downregulation is eased with tumor progression and proliferation, as well as metastasis [66,67]. Important evidence shows how it actively modulates important oncogenic pathways, like Wnt/β-catenin, mTOR (mammalian target of rapamycin), and hypoxia-related signaling [66]. In addition to mTOR, miR-199a-3p negatively regulates STAT3, another critical molecule implicated in Osteosarcoma pathogenesis. The suppression of STAT3 by miR-199a-3p leads to reduced migratory and invasive capabilities of Osteosarcoma cells [27,67]. Moreover, miR-199a-3p actively inhibits the Wnt/β-catenin pathway, thus decreasing the transcriptional activation of genes that are responsible for cell cycle progression as well as migration [66]. miR-199a actively regulates hypoxia-inducible factor-1α (HIF-1α) as well as VEGF. Both factors play an important role in supporting angiogenesis [68]. The restoration importantly reduces angiogenesis (Figure 6). This reduction limits the vascular support that is necessary for tumor growth as well as metastasis. Researchers have conclusively identified that miR-199a-3p acts as an important regulator of high levels of chemoresistance in Osteosarcoma by directly targeting adenylate kinase 4 (AK4) [69]. Many studies indicate that chemoresistant Osteosarcoma cells overexpress AK4, an important enzyme that plays an important role in cellular energy homeostasis and stress response. Lei et al. demonstrated that miR-199a-3p downregulates AK4 expression, thereby impairing the metabolic adaptability of tumor cells and enhancing their sensitivity to chemotherapeutic agents [69]. The suppression of AK4 by miR-199a-3p disrupts cellular tolerance to metabolic stress and reduces drug resistance, highlighting its role in overcoming multi-drug resistance.

**miR-126** is a well-documented tumor suppressor in Osteosarcoma, with its downregulation being strongly related to rapid tumor development, metastasis, and a poor patient prognosis [70,71,72,73]. Its suppressive actions are generally achieved through the regulation of angiogenesis, invasion, and metastasis, targeting major carcinogenic pathways and substances. Work by Jiang et al. and Wang et al. established that miR-126 preferentially targets disintegrin and metalloproteinase 9 (ADAM9) [74,75], a protease known to enhance tumor invasion and metastasis by disrupting the extracellular matrix and aiding tumor cell motility. By lowering ADAM9 expression, miR-126 lowers Osteosarcoma cell motility and invasiveness, limiting the tumor’s spreading potential [74,75]. Furthermore, miR-126 impacts important signaling pathways involved in tumor cell survival and migration. Zhang et al. found that miR-126 lowers the PI3K/AKT pathway, a crucial regulator of cell growth and anti-apoptotic signaling [71]. By targeting components of this system, miR-126 promotes apoptosis and decreases Osteosarcoma cell proliferation, proving its tumor-suppressive effect. Clinically, reduced miR-126 levels relate to advanced tumor stages, greater metastatic burden, and shorter overall survival in Osteosarcoma patients [70]. These findings identify miR-126 as not merely a tumor suppressor but also a potential biomarker for predicting disease progression and patient outcomes.

**miR-1** is a strong tumor suppressor in Osteosarcoma, with its downregulation directly connected with rapid tumor development, angiogenesis, and poor prognosis [76,77]. miR-1 exerts its tumor-suppressive activities through the modulation of key pathways involved in cell cycle control, apoptosis, and angiogenesis. One of the major strategies by which miR-1 decreases tumor growth is its direct targeting of VEGFA, a critical regulator of angiogenesis [77]. Niu et al. demonstrated that miR-1 lowers VEGFA expression, thereby reducing the formation of new blood vessels required to sustain tumor growth [77]. By blocking angiogenesis, miR-1 effectively starves Osteosarcoma cells of nourishment and oxygen, restricting their proliferation and invasiveness. In addition to its role in angiogenesis, Wang et al. discovered that miR-1 regulates the cell cycle downregulating MET (hepatocyte growth factor receptor) [78] which is often overexpressed in Osteosarcoma and contributes to tumor cell proliferation and migration [79]. The suppression of MET by miR-1 produces G0/G1 cell cycle arrest and inhibits cell migration, showing its dual role in regulating tumor growth and metastasis [78,79]. Preclinical research confirms these findings, indicating that miR-1 restoration drastically lowers tumor growth and metastatic potential in xenograft models [76]. Consistently, its expression levels are considerably lower in Osteosarcoma tissues compared to normal bone, with this downregulation correlating with advanced disease stages and worse prognosis [76].

**Figure 7:**
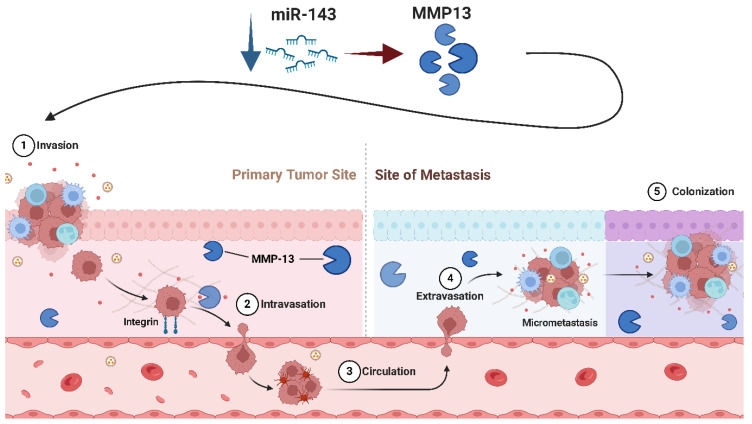
miR-143 plays a crucial role in suppressing the invasive and metastatic potential of osteosarcoma cells. When downregulated, it does not control the expression of MMP-13, a key enzyme involved in extracellular matrix degradation and tumor cell migration. Elevated MMP-13 expression in Osteosarcoma is strongly associated with enhanced metastatic behavior, facilitating tumor cell invasion into surrounding tissues.

**miR-143** operates as a critical tumor suppressor in Osteosarcoma, with its downregulation directly associated with greater tumor aggressiveness, metastatic progression, and resistance to apoptosis [80,81]. A crucial mechanism supporting its tumor-suppressive impact is its potential to regulate apoptosis through the direct targeting of BCL-2, a prominent anti-apoptotic protein commonly overexpressed in Osteosarcoma [82,83]. By lowering BCL-2 expression, miR-143 restores apoptotic pathways, sensitizing Osteosarcoma cells to programmed cell death [83]. This influence is particularly obvious in chemotherapeutic circumstances, when the restoration of miR-143 boosts the efficacy of therapies by enhancing apoptotic rates. In addition to inducing apoptosis, miR-143 performs a vital function in reducing the invasive and metastatic potential of Osteosarcoma cells. This is achieved by the control of MMP-13, a key enzyme involved in extracellular matrix disintegration and the promotion of cell migration [80]. Elevated levels of MMP-13 are intricately connected with more metastatic behavior (Figure 7), and miR-143 directly downregulates this enzyme to limit Osteosarcoma cell invasion [80]. Beyond its influence on apoptosis and metastasis, miR-143 affects cellular proliferation by targeting signaling pathways such as the ERK/MAPK axis, which is typically hyperactivated in Osteosarcoma [84]. By blocking the ERK pathway, miR-143 reduces pro-growth signaling cascades, resulting in reduced tumor cell proliferation and an increased propensity for cell cycle arrest. A meta-analysis supports the suppressive role of miR-143 in Osteosarcoma. The analysis shows that restoration of miR-143 expression leads to reduced tumor cell proliferation, increased apoptosis, and decreased invasive potential. These results suggest that miR-143 may serve as a promising therapeutic target, although further validation is needed to determine its full clinical applicability. Several studies have shown that miR-143 is significantly downregulated in Osteosarcoma tissues compared with normal bone [85]. This downregulation is associated with increased tumor proliferation, increased metastatic potential—particularly lung metastasis—and decreased overall survival. Mechanistically, the loss of miR-143 leads to increased pro-invasive factors such as MMP-13, contributing to the increased migration and invasion of tumor cells [80,86]. Furthermore, clinical data suggest that lower miR-143 expression correlates with a worse prognosis, positioning it as a potential biomarker for Osteosarcoma progression and therapeutic response [87,88].

**miR-145** is a well-characterized tumor suppressor in Osteosarcoma, with its downregulation significantly related to faster tumor development, better metastatic potential, and chemoresistance [89,90]. The tumor-suppressive effects of miR-145 are associated with its propensity to target genes involved in cell proliferation, motility, and invasion. A significant function of miR-145 involves the regulation of cell cycle progression through the targeting of E2F3, a transcription factor that activates genes crucial for the transition from the G1 to S phase [91,92]. By downregulating E2F3, miR-145 accelerates G1 phase arrest, therefore limiting cellular development. In addition to its impact on proliferation, miR-145 demonstrates substantial anti-metastatic capabilities [93]. This suppressive role is further attributed to its regulation of MMP16 and CDK6, two critical factors in Osteosarcoma progression [93,94]. The downregulation of miR-145 leads to the overexpression of MMP16, which increases extracellular matrix degradation and promotes tumor invasion. In addition, the loss of miR-145 results in increased CDK6, a key kinase that facilitates G1/S transition, thereby accelerating uncontrolled cell proliferation. Taking together, these molecular alterations contribute to the aggressive nature of Osteosarcoma, highlighting that miR-145 is a key regulator of tumor progression and a potential therapeutic target.

**Figure 8:**
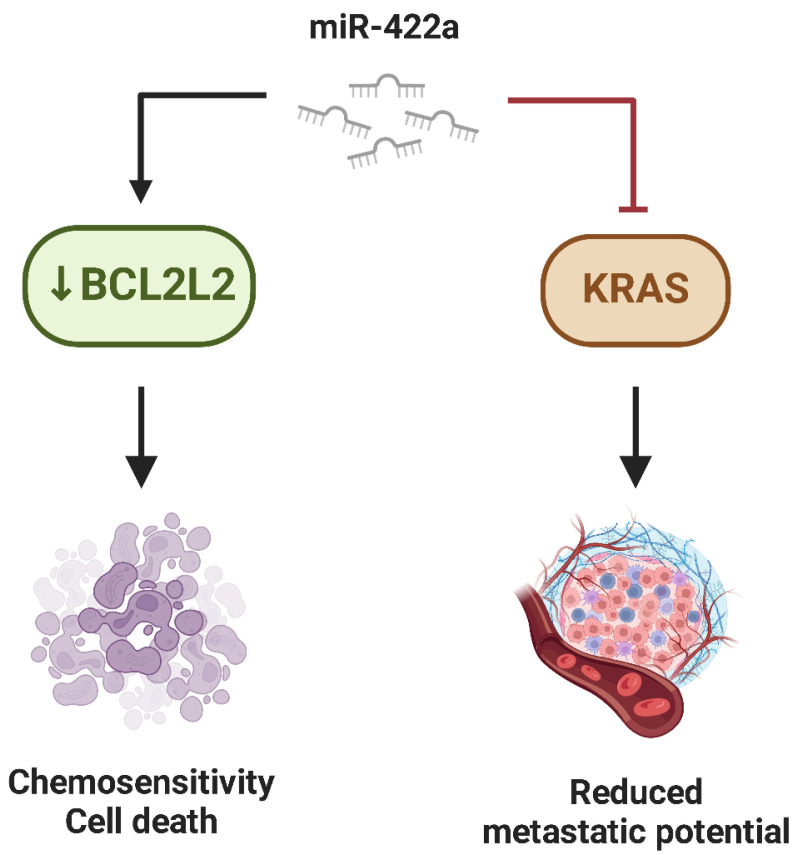
The dual role of miR-422a in Osteosarcoma. **Left:** the downregulation of BCL2L2 enhances tumor sensitivity to chemotherapy, leading to increased cell death. **Right:** the inhibition of KRAS suppresses the migratory and invasive potential of osteosarcoma cells.

Similarly, **miR-422a** is regarded as a tumor suppressor in Osteosarcoma, with its downregulation directly connected to improved tumor formation, resistance to apoptosis, and increased metastatic potential [95,96]. A significant way miR-422a exerts its tumor-suppressive effects is by targeting BCL2L2, an anti-apoptotic gene that maintains tumor cell survival. By downregulating BCL2L2, miR-422a restores the balance between pro-apoptotic and anti-apoptotic signals, thereby improving the sensitivity of Osteosarcoma cells to programmed cell death [96]. Additionally, miR-422a influences metastatic potential by lowering KRAS, a crucial component of the RAS signaling cascade. The inhibition of KRAS expression resulted in lower migratory and invasive capacities of Osteosarcoma cells [96] (Figure 8).

**miR-449c** operates as a strong tumor suppressor in Osteosarcoma, with its downregulation directly related to accelerated tumor proliferation, invasion, and resistance to apoptosis [27,97]. One of the key targets of miR-449c is c-Myc, a well-documented oncogene that promotes tumor growth. By lowering c-Myc expression, miR-449c lowers cellular proliferation and mitigates tumor aggressiveness [97]. In addition, miR-449c is downregulated in Osteosarcoma tissues, probably due to promoter hypermethylation. One study associated low levels of miR-449c with a worse prognosis, highlighting its potential as a biomarker and therapeutic targe [97].

Similarly, **miR-125b** works as a tumor suppressor in Osteosarcoma, with its downregulation closely connected to enhanced tumor development, resistance to apoptosis, and heightened metastatic potential [98,99]. Through the direct suppression of BCL-2 expression, miR-125b reactivates apoptotic pathways, hence boosting tumor cell sensitivity to apoptosis [98]. Beyond its involvement in apoptosis regulation, miR-125b affects tumor growth by targeting genes involved in the cell cycle, such as c-Raf, a serine/threonine kinase that initiates the MAPK/ERK signaling cascade [100]. The reduction of c-Raf by miR-125b resulted in reduced cell proliferation and increased susceptibility for cell cycle arrest [101].

**Figure 9:**
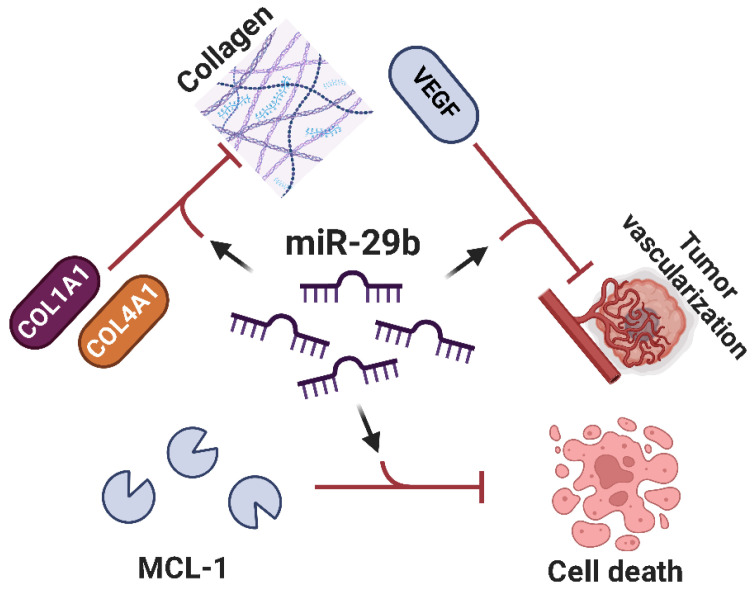
Tumor-suppressive role of miR-29b in Osteosarcoma. miR-29b downregulation is associated with enhanced tumor proliferation, angiogenesis, and metastasis. By targeting COL1A1 and COL4A1, miR-29b inhibits extracellular matrix remodeling, limiting tumor invasion. Additionally, it suppresses VEGF-mediated angiogenesis and promotes apoptosis by downregulating anti-apoptotic proteins such as MCL1 and BCL-2.

**miR-29b** represents a crucial tumor suppressor in Osteosarcoma, with its downregulation strongly related to accelerated tumor proliferation, angiogenesis, and metastatic potential [102,103,104,105,106,107]. One key method via which miR-29b exerts its tumor-suppressive effects is via the control of collagen synthesis pathways [108]. miR-29b targets and suppresses numerous genes involved in the manufacture of extracellular matrix proteins, including COL1A1 and COL4A1 [108]. By inhibiting the excessive creation of these collagens, miR-29b curtails the structural remodeling necessary for tumor invasion and metastasis. Additionally, miR-29b regulates tumor-induced angiogenesis by blocking VEGF, effectively denying the tumor of oxygen and nutrients required for development and dispersion [103]. Aside from its effects in invasion and angiogenesis, miR-29b promotes apoptosis by targeting anti-apoptotic proteins such as MCL1 (myeloid cell leukemia 1) and BCL-2, therefore repairing damaged apoptotic pathways (Figure 9) [108]. Given its multifaceted tumor-suppressive functions, miR-29b emerges as a promising therapeutic target in Osteosarcoma. Restoring miR-29b expression could counteract tumor progression by simultaneously inhibiting extracellular matrix remodeling, angiogenesis, and evasion of apoptosis, enhancing its therapeutic potential in Osteosarcoma treatment.

Lastly, **miR-154-5p** is known as a tumor suppressor in Osteosarcoma, with its downregulation considerably linked with improved tumor proliferation, motility, and resistance to apoptosis [109,110]. A significant role of miR-154-5p is its regulation of cell cycle progression through the targeting of genes such as Cyclin E1 (CCNE1) and CDK2, which are vital in regulating the G1/S phase transition [110]. By inhibiting these molecules, miR-154-5p promotes G1 phase arrest, ultimately preventing cell division and proliferation [110]. Its tumor-suppressive function is further supported by studies in Osteosarcoma models, where the restoration of miR-154-5p reduces tumor growth and metastatic spread [110].

### 2.4. miRNAs in Ewing’s Sarcoma

Ewing’s sarcoma (ES) is an aggressive cancer that primarily affects children, adolescents, and young adults, making it the second most common primary bone tumor in these age groups. While it usually develops in bones, it can also arise in soft tissues. The disease is driven by a specific chromosomal translocation that creates the EWS-FLI1 fusion gene, formed by the rearrangement of the EWS gene on chromosome 22 and an ETS family gene, usually FLI1, on chromosome 11 [111]. This fusion protein functions as an abnormal transcription factor, disrupting the expression of numerous genes, including miRNAs [112,113].

**Figure 10:**
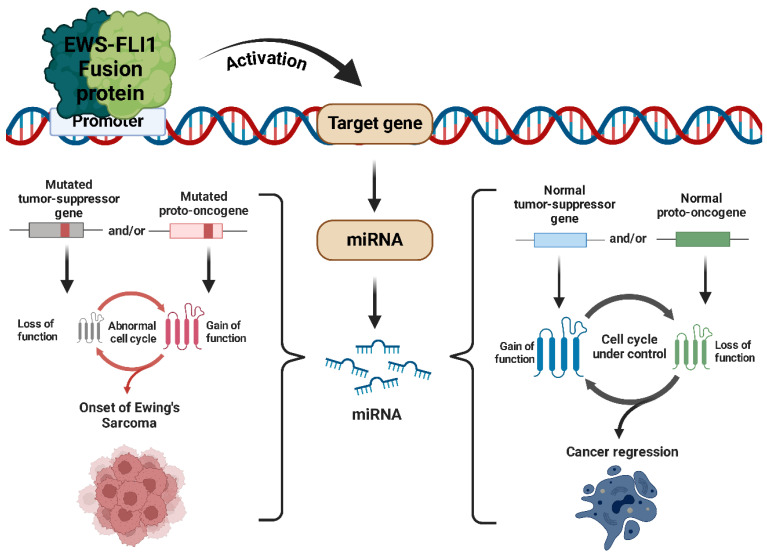
The EWS-FLI1 fusion oncoprotein exerts regulatory control over miRNAs, which play a dual role in Ewing’s sarcoma. Certain miRNAs function as oncogenic enhancers by interacting with multiple oncogenes, amplifying tumor-promoting effects, while others act as tumor suppressors, counteracting oncogenic signaling. Understanding this intricate regulatory network is crucial for developing targeted therapeutic strategies.

The dysregulation of miRNAs by EWS-FLI1 affects multiple signaling pathways, including those governing cell cycle regulation, apoptosis, and metastatic potential (Figure 10). For instance, the EWS-FLI1-mediated suppression of miR-145 leads to the upregulation of IGF1R, a crucial receptor implicated in ES cell survival and metastasis. Similarly, the downregulation of let-7 miRNA family members enhances the expression of HMGA2, a chromatin remodeling factor associated with increased tumor aggressiveness. Moreover, the modulation of miR-34a by EWS-FLI1 disrupts p53 signaling, further contributing to the evasion of apoptosis and unchecked cellular proliferation [114]. Given the profound impact of EWS-FLI1 on miRNA regulation, miRNAs are being actively investigated as both biomarkers and therapeutic targets in ES. Strategies aimed at restoring tumor-suppressive miRNAs or inhibiting oncogenic miRNAs are currently being explored, with promising preclinical results. The use of miRNA mimics, antagomiRs, or small molecules targeting the EWS-FLI1–miRNA axis holds potential for novel therapeutic interventions that could improve patient outcomes [115].

### 2.5. Oncogenic miRNAs in Ewing’s Sarcoma

Multiple miRNAs with oncogenic properties have been found to play a role in Ewing’s sarcoma, promoting tumor progression, metastatic dissemination, and resistance to chemotherapy.

**Figure 11:**
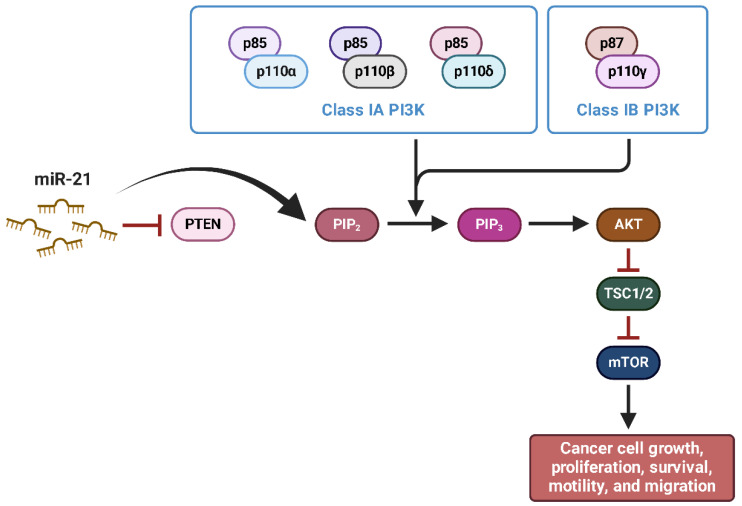
miR-21 overexpression contributes to Ewing’s sarcoma progression by enhancing cell proliferation, migration, invasion, and resistance to apoptosis. A key target of miR-21 in ES is the tumor suppressor PTEN, a negative regulator of the PI3K/AKT signaling pathway. By repressing PTEN, miR-21 drives PI3K/AKT hyperactivation, promoting cell survival and unchecked tumor growth. These findings underscore miR-21 as a potential therapeutic target for restoring tumor suppressive mechanisms in ES.

**miR-21** is one of the most thoroughly researched oncogenic microRNAs, with its overexpression linked in the advancement of numerous malignancies, including ES [116,117]. In this tumor type, miR-21 has been found to increase cell proliferation, migration, invasion, and resistance to apoptosis by targeting important tumor suppressor genes and dysregulating molecular pathways that are essential for maintaining cellular homeostasis [116,117]. One of the key targets of miR-21 in ES is PTEN, a well-established tumor suppressor that adversely regulates the PI3K/AKT signaling pathway [118]. The suppression of PTEN expression by miR-21 results in the hyperactivation of the PI3K/AKT pathway, which in turn leads to an increase in cell survival, improved proliferation, and a reduction in apoptosis (Figure 11) [118].

**miR-181a** has been identified as a deregulated microRNA in ES, although its precise role in tumor progression has yet to be fully elucidated [114,115]. A comprehensive analysis of miRNA expression profiles in ES tissue samples revealed that miR-181a is significantly upregulated compared with normal mesenchymal stromal cells (MSCs) [116]. This suggests that miR-181a may contribute to the oncogenic phenotype of ES by modulating key signaling pathways involved in cell survival, proliferation, and metastasis. Bioinformatic analysis indicates that BCL-2, is a direct target of miR-181a, implicating this miRNA in the regulation of apoptosis resistance in ES cells [114]. Although miR-181a has been implicated in multiple tumor processes, its specific oncogenic role in ES remains partially characterized.

**Figure 12:**
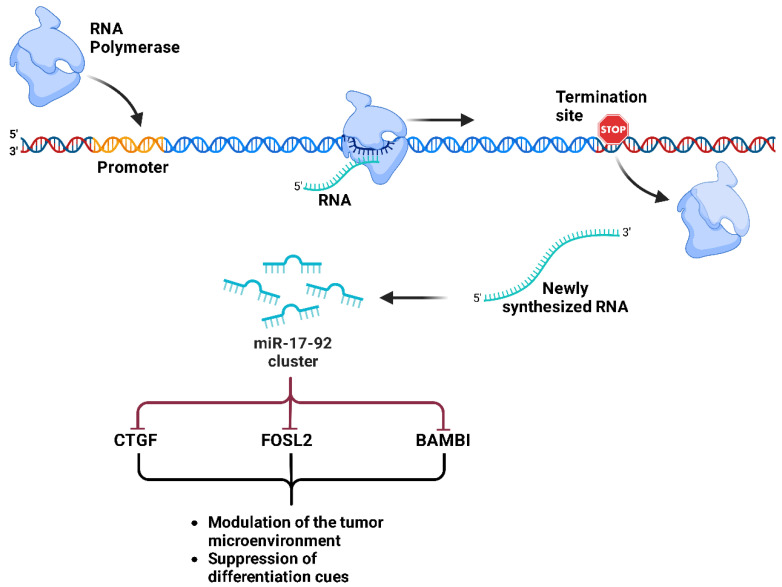
The miR-17-92 cluster acts as a central oncogenic hub in Ewing’s sarcoma, directly activated by the EWS-FLI1 fusion oncoprotein. By targeting key components of the TGFB/BMP signaling pathway, including CTGF, FOSL2, and BAMBI, miR-17-92 modulates the tumor microenvironment and suppresses differentiation signals, thereby promoting tumorigenesis. These findings suggest that therapeutic inhibition of miR-17-92 may restore tumor-suppressive pathways and represent a potential strategy for targeted treatment.

The **miR-17-92** cluster is a well-characterized group of oncogenic miRNAs that plays a crucial role in the progression of ES, regulated mainly by the oncogenic fusion protein EWS-FLI1 [119]. EWS-FLI1 has been shown to directly activate miR-17-92 transcription, leading to multiple tumor suppression pathways. The TGFB/BMP signaling pathway has been identified as a key downstream target of miR-17-92 in ES [119]. Furthermore, miR-17-92 negatively regulates key genes in these pathways, including CTGF, FOSL2 and BAMBI, contributing to ES tumorigenesis through the modulation of the tumor microenvironment and the suppression of differentiation cues (Figure 12) [119]. These results highlight that miR-17-92 is a central oncogenic hub in ES and suggest that therapeutic inhibition of this miRNA cluster may restore tumor suppressive pathways.

**miR-125b** has been identified as a key regulator in ES, playing a crucial role in chemoresistance, cell proliferation, and survival through different mechanisms [120,121,122]. miR-125b has been shown to be upregulated in doxorubicin-resistant ES cells and tumors that have survived chemotherapy, suggesting its involvement in drug resistance [122]. Mechanistically, miR-125b suppresses pro-apoptotic mediators, such as p53 and BAK, thereby increasing cell survival and reducing sensitivity to chemotherapeutic agents such as doxorubicin, vincristine, and etoposide [122]. Reducing miR-125b levels in resistant ES cells restored chemosensitivity, highlighting its potential as a therapeutic target to overcome drug resistance. In contrast, a tumor suppressor role for miR-125b in ES has been suggested, with reports showing that miR-125b is downregulated in ES tissues compared with normal bone tissue [120]. Functional assays have shown that the restoration of miR-125b expression inhibits proliferation, migration, and invasion and induces apoptosis in ES cells [120]. This antitumor function appears to be mediated by the direct inhibition of PIK3CD, a key regulator of the PI3K/AKT/mTOR signaling pathway known to promote tumorigenesis [120]. These conflicting results suggest that miR-125b may have a dual role in ES, depending on the tumor context and specific regulatory networks. While it promotes chemoresistance through the inhibition of p53 and BAK, it may also suppress tumor growth by inhibiting the PI3K/AKT/mTOR pathway. Further research is needed to elucidate these opposing functions and explore miR-125b as a potential therapeutic target in the treatment of ES.

### 2.6. Tumor Suppressor miRNAs in Ewing’s Sarcoma

In opposition to oncogenic miRNAs, several others serve as tumor suppressors in ES, inhibiting both tumor development and metastatic dissemination.

**miR-22** has been identified as a tumor suppressor miRNA in ES that functions in opposition to the oncogenic program driven by the EWS/FlI1 fusion oncoprotein [123]. A study showed that EWS/FlI1 represses miR-22 expression, and its restoration leads to significant inhibition of tumor cell proliferation, clonogenic growth, and anchorage-independent survival [123]. This highlights its potential therapeutic role in counteracting ES progression. Mechanistically, miR-22 exerts its antitumor effects by directly targeting histone demethylase KDM3A (also known as JMJD1A), which is known to promote oncogene expression and drive tumorigenesis. KDM3A is overexpressed in ES, and its deletion inhibits tumor cell growth both in vitro and in xenograft models, demonstrating its crucial role in the pathophysiology of ES [123]. KDM3A suppression by miR-22 leads to increased levels of the repressive histone H3K9me2, resulting in the downregulation of pro-oncogenic factors such as cyclin D1, IGF-1R, and ETS1. These results establish miR-22 as a critical regulator of epigenetic modifications in ES, underscoring its therapeutic potential.

**miR-30a** has emerged as a key tumor suppressor miRNA in ES, with growing evidence supporting its involvement in inhibiting tumor development, chemoresistance, and metastasis [124,125,126]. miR-30a-5p plays a crucial role in ES by modulating two important therapeutic targets: EWS-FLI1 and CD99 [125]. EWS-FLI1, an aberrant transcription factor that drives ES oncogenesis, has been shown to negatively regulate miR-30a-5p expression. In vivo and in vitro experiments have shown that the inhibition of EWS-FLI1 leads to a significant reduction in CD99 protein levels despite minimal changes in its mRNA expression, suggesting a post-transcriptional regulatory mechanism involving microRNAs [125]. Among the affected microRNAs, miR-30a-5p directly targets the 3′UTR of CD99, thus reducing its expression. Functional analyses found that reintroduction of miR-30a-5p into ES cells results in decreased proliferation and invasion, highlighting its role as a tumor suppressor. These results suggest that restoration of miR-30a-5p expression could serve as a promising therapeutic approach to counteract the progression of ES by disrupting the EWS-FLI1/CD99 oncogenic axis [125].

**Figure 13:**
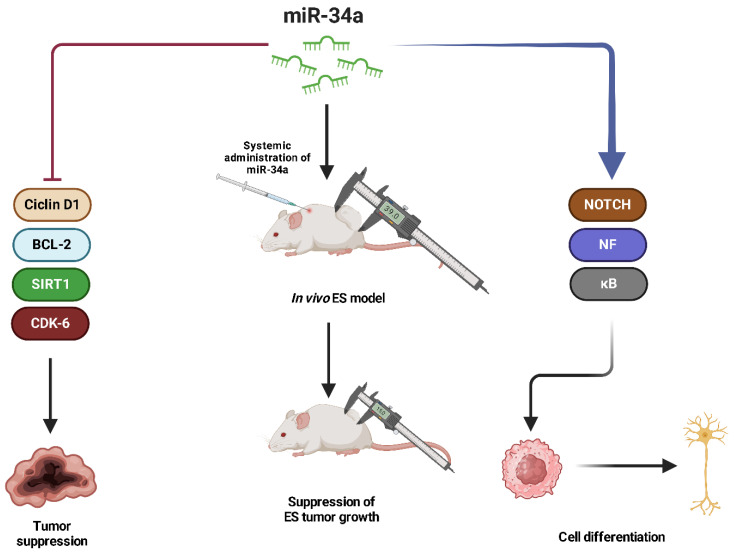
miR-34a functions as a critical tumor suppressor in Ewing’s sarcoma, regulating apoptosis, cell cycle progression, and chemoresistance through the suppression of key oncogenic regulators such as cyclin D1, BCL-2, SIRT1, and CDK6. miR-34a also modulates the NOTCH-NF-κB axis, promoting neural differentiation and reducing tumorigenicity. In vivo studies demonstrate that the systemic delivery of miR-34a significantly inhibits tumor growth.

**miR-34a** has been established as a tumor suppressor in ES, playing a crucial role in regulating apoptosis, cell cycle progression, and chemoresistance [127]. Multiple studies have demonstrated that high miR-34a expression is correlated with improved prognosis and better event-free and overall survival in ES patients [127,128,129,130,131]. Its expression is significantly lower in metastatic ES compared to localized tumors, suggesting a role in disease progression. miR-34a exerts its tumor suppressor function by targeting key oncogenic regulators such as cyclin D1, BCL-2, SIRT1, and CDK6, which are involved in cell proliferation and survival [130]. Restoring miR-34a levels in ES cells leads to decreased proliferation, increased apoptosis, and increased sensitivity to chemotherapy treatment with doxorubicin and vincristine, supporting its potential as a therapeutic target [127,130,131]. In addition, miR-34a has been implicated in the regulation of the NOTCH-NF-κB signaling axis, where its upregulation promotes the neural differentiation of ES cells, thereby reducing tumorigenicity. In vivo studies have shown that the systemic administration of bioengineered miR-34a-5p significantly suppresses ES tumor growth in xenograft models, further highlighting its potential in RNA-based therapies (Figure 13) [127]. Given its strong association with prognosis and therapeutic response, miR-34a represents a promising biomarker and therapeutic target in ES.

**miR-124** has been discovered to be a tumor suppressor in ES, affecting pathways that are crucial for cell cycle regulation, EMT, and apoptosis [132,133,134]. Studies have shown that miR-124 expression is significantly downregulated in ES tissues and cell lines compared to normal tissues, with even lower levels in metastatic lesions [132,133,134]. miR-124 directly targets SLUG (SNAI2), a key regulator of EMT, leading to the upregulation of E-cadherin and the suppression of mesenchymal markers such as Vimentin and N-cadherin [134]. The same study also highlighted the role of epigenetic regulation in miR-124 suppression, as hypermethylation of the promoter region of miR-124 contributes to its downregulation in ES [134]. Treatment with DNA methylation inhibitors has been shown to restore miR-124 expression, resulting in reduced proliferation, migration, and invasion. In addition, miR-124 has been implicated in a novel regulatory axis involving the METTL3/m6A-modified MALAT1/miR-124-3p/CDK4 pathway, where METTL3 upregulates MALAT1, which in turn sponges off miR-124, preventing it from inhibiting CDK4 [132]. This mechanism promotes tumor progression and metastasis, reinforcing the suppressive role of miR-124 in ES.

The **miR-145** works as a tumor suppressor in ES, targeting the EWS-FLI1 fusion oncogene [135,136,137]. Studies have shown that EWS-FLI1 directly represses miR-145 expression and, conversely, miR-145 targets the EWS-FLI1 fusion oncogene, creating a negative feedback loop that regulates tumor growth [137]. In addition, miR-145 plays a crucial role in suppressing EMT in ES by targeting key transcription factors such as SOX2, KLF4 and OCT4, which are involved in maintaining tumor stemness properties [135]. The overexpression of miR-145 in ES cell lines has been shown to inhibit anchorage-independent migration, invasion, and growth, reinforcing its role in preventing metastasis. Finally, miR-145 modulates the expression of mesenchymal markers such as vimentin, N-cadherin, and snail, while regulating E-cadherin, thereby reducing the metastatic potential of ES cells [135].

**miR-193b** is significantly downregulated in ES cells compared with normal hMSCs, largely due to repression by the oncogenic fusion protein EWS-FLI1 [138]. The restoration of miR-193b expression in ES cell lines significantly inhibits proliferation and clonogenic growth, indicating its tumor suppressor properties. Importantly, miR-193b directly targets the ErbB4 oncogene, a known promoter of tumor progression and metastasis in ES. The study confirmed that miR-193b downregulates ErbB4 expression, leading to anchorage-independent growth reduction and decreased tumorigenic potential [138]. Furthermore, the inhibition of ErbB4 expression by shRNA mimics the tumorigenic effects of miR-193b, further solidifying the link between miR-193b and the regulation of key oncogenic pathways in ES [138].

**miR-185** has been shown to be significantly downregulated in ES cells, and its overexpression leads to reduced tumor cell viability, the suppression of migration, and increased apoptosis [139]. miR-185 exerts its tumor-suppressive function by targeting the PI3K/AKT/mTOR and Wnt/β-catenin pathways, both of which are crucial for ES cell survival and metastasis. The increase in miR-185 in ES cells led to the downregulation of phosphorylated AKT and mTOR, effectively impairing pro-survival signaling [139]. In addition, miR-185 suppressed Wnt3a and β-catenin, leading to reduced mesenchymal characteristics and metastatic potential. Finally, miR-185 was found to directly target E2F6, a transcription factor involved in cell cycle progression and tumorigenesis. Restoring E2F6 levels restored the tumorigenic effects of miR-185, indicating that the miR-185/E2F6 axis is crucial for tumor regulation in ES [139].

**Figure 14:**
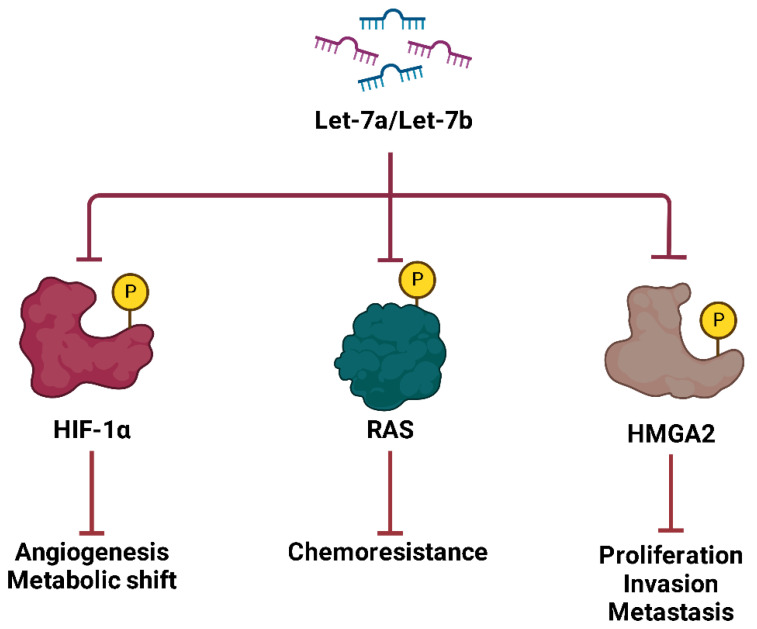
The administration of Let-7a and Let-7b in in vivo models of ES has been shown to inhibit key oncogenes such as RAS, HIF-1α, and HMGA2, leading to a significant suppression of tumorigenic properties such as angiogenesis, metabolic reprogramming, and resistance to chemotherapeutic treatments, as well as a marked decrease in tumor cell proliferation, invasiveness, and metastatic potential.

Studies have shown that **Let-7a** and **Let-7b** are significantly downregulated in ES tumors, and their loss is associated with increased relapse rates and poor prognosis [114,140]. Let-7 showed direct targets oncogenes such as RAS, HIF-1α and HMGA2, thus inhibiting tumor growth and metastasis (Figure 14). In ES, repression of Let-7 leads to upregulation of HIF-1α, which in turn promotes the expression of EWS-FLI1, contributing to the aggressive nature of the disease [114,140]. In addition, in vivo experiments showed that systemic administration of synthetic Let-7a in ES xenograft models significantly reduced tumor growth, suggesting a potential therapeutic strategy to target the downregulation of Let-7 in ES [114,140]. Given its strong tumor suppressor function, strategies to restore Let-7 expression or target its downstream oncogenic pathways could offer promising therapeutic approaches for the treatment of ES.

**miR-138** is significantly downregulated in ES cell lines compared with hMSCs and its expression is inversely correlated with the levels of focal adhesion kinase (FAK), a key factor in cancer cell proliferation and invasion [141]. miR-138 directly targets FAK, leading to a reduction in cell adhesion, migration, and metastatic potential in ES. In vivo experiments confirmed that the overexpression of miR-138 significantly suppresses distant metastasis formation, further supporting its tumor suppressive role [141]. These results establish that miR-138 is a key regulator of ES progression, where its loss contributes to increased tumor aggressiveness and metastatic potential. The restoration of miR-138 or its downstream effectors, such as FAK, could be a promising therapeutic strategy for the treatment of ES.

**miR-107** exerts its tumor-suppressive effects by directly targeting hypoxia-inducible factor-1β (HIF-1β), a key regulator of tumor cell metabolism, angiogenesis, and survival under hypoxic conditions [142]. The overexpression of miR-107 in ES cell lines leads to cell cycle arrest in G1 phase and increases apoptosis, suggesting its role in inhibiting tumor progression. In addition, miR-107 reduces the ability of ES cells to form vascular-like structures, implicating it in suppressing angiogenesis and metastatic potential [142]. These results establish that miR-107 is a critical regulator of tumor growth and vascularization in ES and that its downregulation contributes to increased tumor aggressiveness. Given its role in targeting HIF-1β and disrupting oncogenic pathways, restoring miR-107 expression could serve as a promising therapeutic strategy for the treatment of ES.

**miR-708** has been identified as a key regulator of tumor progression and chemoresistance in ES, acting through the modulation of EYA3 (Eyes Absent Homolog 3), a tyrosine phosphatase involved in DNA damage repair [113,143]. EWS-FLI1 represses miR-708 expression, leading to an increase in EYA3, which in turn increases tumor cell survival and resistance to chemotherapeutic agents. It has been reported that restoring miR-708 expression in ES cells reduces EYA3 levels, impairs DNA repair mechanisms, and sensitizes tumor cells to DNA-damaging agents such as etoposide and doxorubicin [113]. This suggests that miR-708 functions as a tumor suppressor, counteracting the effects of EYA3-mediated chemoresistance. Furthermore, the modulation of miR-708 has been implicated in EWS-FLI1-driven metastasis. While previous studies suggest that miR-708 generally suppresses tumor invasion, its role in ES remains complex. In some contexts, forced overexpression of miR-708 increased MMP2 expression and promoted invasion, highlighting a dual regulatory role in tumor aggressiveness [143].

In Ewing’s sarcoma cells, **miR-199a-3p** is significantly downregulated, whereas its overexpression has been shown to inhibit proliferation, migration, and invasion [144,145]. Mechanistically, miR-199a-3p functions in the context of the TUG1-miR-199a-3p-MSI2 axis, in which the long noncoding RNA TUG1 acts as a competing endogenous RNA (ceRNA) to sequester miR-199a-3p, thereby increasing the expression of the oncogenic protein MSI2 [144]. In addition, the exosomal transfer of miR-199a-3p-enriched vesicles from CD99-deficient ES cells has been shown to reduce tumor aggressiveness, suppress cell migration, and promote neural differentiation, further enhancing its antitumor potential [145]. These results suggest that the restoration of miR-199a-3p expression could be a promising therapeutic strategy for ES.

### 2.7. miRNAs in Chondrosarcoma

**Figure 15:**
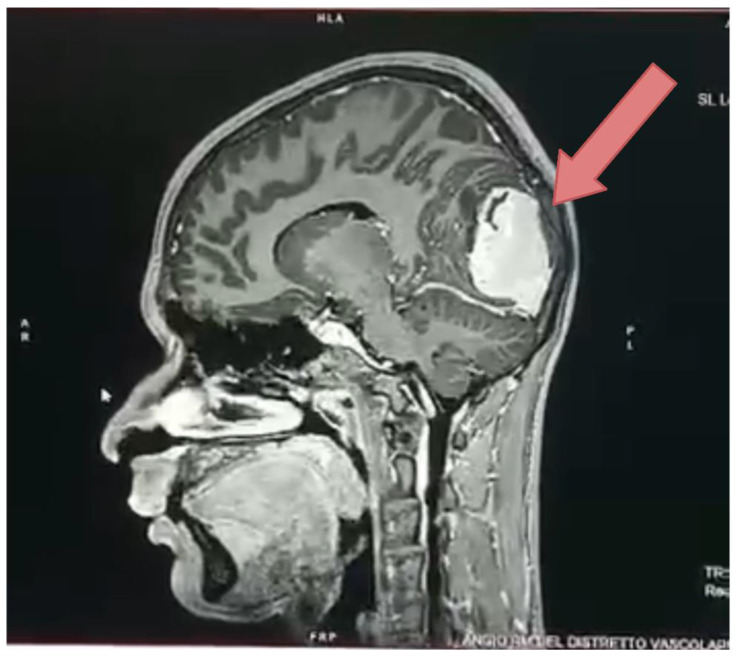
MRI of the brain with contrast enhancement (MDC) and MR angiography of the intracranial arterial circulation revealed a Chondrosarcoma appearing as an isointense mass in the occipital–parasagittal region (as indicated by red arrow), measuring 52 × 53 × 43 mm. The lesion is durally based on the lateral aspect of the confluence of sinuses on the left side, with its inferior margin delineated by the tentorium cerebelli. At its site of attachment, the mass is in close adherence to the left transverse sinus, near the confluence of sinuses, causing approximately 8 mm of compression.

Chondrosarcoma is an aggressive malignant bone tumor that primarily affects adults, making it the second most common primary bone malignancy in this age group [146]. Unlike other sarcomas, Chondrosarcoma is characterized by the production of a cartilage matrix (Figure 15) by transformed chondrocytes and is notably resistant to conventional chemotherapy and radiation therapy. As a result, surgical resection remains the primary treatment option [147].

The molecular pathogenesis of Chondrosarcoma is highly complex, involving a range of genetic and epigenetic alterations. Among these, miRNAs have emerged as key regulatory molecules, influencing various aspects of tumor biology. miRNAs in Chondrosarcoma can function as either oncogenes or tumor suppressors, affecting critical cellular processes such as proliferation, apoptosis, and metastasis [21]. Given their role in tumor progression, miRNAs are increasingly being explored as potential biomarkers and therapeutic targets in Chondrosarcoma.

### 2.8. Oncogenic miRNAs in Chondrosarcoma

**miR-181a** plays a critical role in Chondrosarcoma pathogenesis, enabling tumor development, angiogenesis, and metastasis through the regulation of pivotal molecular pathways [148,149]. Its expression is significantly upregulated in high-grade Chondrosarcoma and is induced by hypoxia, a condition that increases tumor aggression. miR-181a exerts its oncogenic effects by targeting RGS16, a negative regulator of CXCR4 signaling, thereby amplifying CXCR4-mediated pathways involved in increasing VEGF and MMP1 [149]. These molecular alterations facilitate increased angiogenesis and metastatic potential. The inhibition of miR-181a in preclinical models resulted in the restoration of RGS16 expression and the reduction of angiogenesis, growth, and lung metastasis of the tumor [149]. In addition, the overexpression of miR-181a was confirmed in Chondrosarcoma tissue samples, supporting its role as a potential biomarker and therapeutic target [148,149].

In Chondrosarcoma, miR-101 operates as an oncogenic miRNA, increasing tumor growth, migration, and invasion by targeting pathways critical for extracellular matrix remodeling and cellular motility [150]. Its overexpression has been related to enhanced tumor aggressiveness and metastatic potential. A main target of miR-101 in Chondrosarcoma is TIMP-3 (tissue inhibitor of metalloproteinases-3), a critical regulator of metalloproteinase activity that preserves extracellular matrix integrity. The downregulation of TIMP-3 by miR-101 has been reported to increase the activity of MMPs, specifically MMP-2 and MMP-9, which in turn facilitate tumor cell invasion and migration [150]. This regulatory interaction is critical, as MMP-2 is known for its ability to degrade extracellular matrix, a key step in cancer invasion and metastasis. Specifically, sphingosine-1-phosphate (S1P), a bioactive sphingolipid, has been shown to inhibit miR-101 expression, restoring TIMP-3 levels and reducing metastatic potential in vitro and in vivo [150].

### 2.9. Tumor Suppressor miRNAs in Chondrosarcoma

In contrast, certain miRNAs function as tumor suppressors in Chondrosarcoma, restraining tumor growth and metastatic spread.

**Figure 16:**
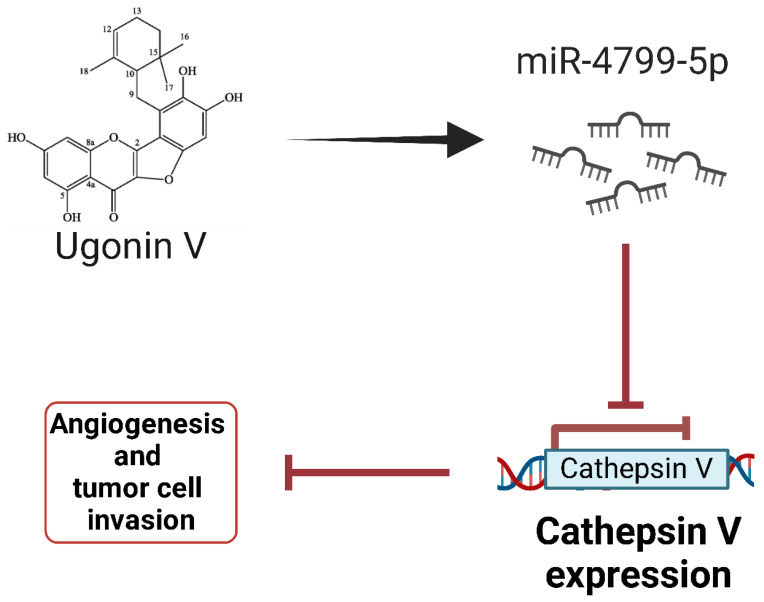
The natural flavonoid compound Ugonin V induces the overexpression of miR-4799-5p, which in turn inhibits the expression of cathepsin V (CTSV), a cysteine protease implicated in tumor progression. This regulatory pathway leads to reduced angiogenesis, cell invasion, and metastasis.

Very recent research has identified **miR-4799-5p** as a crucial regulator in the suppression of Chondrosarcoma metastasis [151]. The natural flavonoid compound Ugonin V has been shown to regulate miR-4799-5p, leading to downregulation of cathepsin V (CTSV), a cysteine protease implicated in tumor progression (Figure 16). Bioinformatic analysis and luciferase reporter assays confirmed that miR-4799-5p directly targets the 3′-UTR of CTSV mRNA, thus inhibiting its expression [151]. Functional studies showed that the overexpression of miR-4799-5p significantly reduced Chondrosarcoma cell migration and invasion, while its inhibition restored CTSV expression and metastatic potential. In vivo models showed that the administration of Ugonin V increased the levels of miR-4799-5p, which correlated with reduced CTSV expression and decreased lung metastasis [151]. These results suggest that miR-4799-5p functions as a tumor suppressor in Chondrosarcoma and could be a potential therapeutic target to inhibit metastasis.

**miR-125b** acts as a tumor suppressor in Chondrosarcoma, inhibiting tumor growth, metastasis, and chemoresistance [152,153]. Indeed, miR-125b is significantly downregulated in Chondrosarcoma tissues and cell lines, particularly in metastatic cases. One of its key mechanisms involves directly targeting ErbB2, a regulator of glucose metabolism, leading to the inhibition of glycolysis and increased chemosensitivity to doxorubicin [153]. In addition, arsenic trioxide (ATO) has been shown to regulate miR-125b expression through DNA demethylation, triggering MET and reducing cell migration and invasion. Finally, miR-125b directly suppresses the transducer and activator of transcription signal 3 (STAT3), a key player in the regulation of EMT and metastasis, further enhancing its tumor suppressor role [152].

**miR-23b** functions as a tumor suppressor in Chondrosarcoma, mainly by modulating the Src-AKT signaling pathway and increasing chemosensitivity to cisplatin [154]. The overexpression of miR-23b leads to the suppression of tumor cell proliferation, migration, and invasion, suggesting its critical role in tumor suppression. A key mechanism by which miR-23b exerts its effects is the direct targeting of Src kinase, an oncogenic protein that regulates cell survival and chemoresistance [154]. The downregulation of miR-23b is associated with increased Src-AKT pathway activity, which contributes to cisplatin resistance in Chondrosarcoma cells [154]. Importantly, the restoration of miR-23b expression resensitizes Chondrosarcoma cells resistant to cisplatin treatment, highlighting its potential as a therapeutic target to overcome chemoresistance.

Some findings highlight that **miR-30a** is a key tumor suppressor in Chondrosarcoma, where its downregulation contributes to disease progression [155,156]. Expression analyses indicate that miR-30a levels are significantly downregulated in Chondrosarcoma tissues and cell lines, correlating with increased tumor aggressiveness. miR-30a directly targets the transcription factors SOX4 and Runx2, both of which are implicated in promoting proliferation, migration and invasion of cancer cells [155,156]. Through SOX4 inhibition, miR-30a suppresses oncogenic signaling pathways that drive tumor growth, while its regulation of Runx2 disrupts key processes of chondrogenesis and metastatic potential. Restoring miR-30a expression in chondrosarcoma cells induces cell cycle arrest, reduces proliferation, and impairs invasive capabilities, further supporting its role as a potential therapeutic target [155,156].

**Figure 17:**
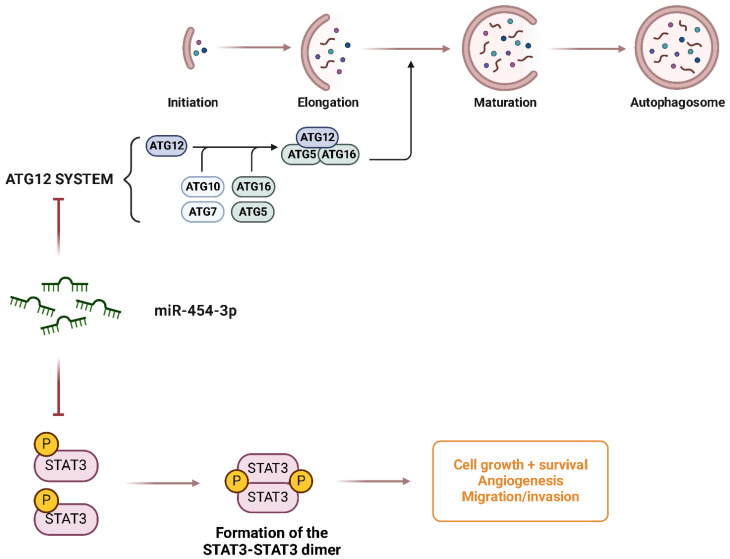
The overexpression of miR-454-3p leads to the inhibition of STAT3 and ATG12, which are involved in molecular pathways promoting Chondrosarcoma cell proliferation and evasion of apoptotic and autophagic mechanisms. By suppressing these pathways, miR-454-3p highlights its potential role in developing a therapeutic strategy aimed at Chondrosarcoma regression.

**miR-454-3p** functions as a tumor suppressor in Chondrosarcoma by inhibiting major oncogenic pathways [149]. The expression of miR-454-3p is significantly downregulated in Chondrosarcoma tissues and cell lines, and its suppression is mediated by the long noncoding RNA HOTAIR, which recruits EZH2 and DNMT1 to promote the DNA methylation of the miR-454-3p promoter, thereby silencing its expression [149]. Functional analysis revealed that miR-454-3p directly targets STAT3 and ATG12, two key regulators of tumor proliferation and autophagy, respectively (Figure 17). By suppressing these targets, miR-454-3p inhibits Chondrosarcoma cell growth, promotes apoptosis, and reduces autophagy, suggesting a critical role in tumor suppression. In vivo experiments have shown that the overexpression of miR-454-3p significantly reduces tumor growth, further supporting its therapeutic potential [149].

The **miR-143/145** cluster plays a vital role in tumor suppression in Chondrosarcoma, where its downregulation is associated with accelerated tumor motility, invasion, and progression [157]. miR-143 and miR-145 have been shown to function as key modulators of FSCN1, an actin-binding protein that increases cell motility, adhesion, and invasion [157]. The downregulation of miR-143/145 in Chondrosarcoma leads to increased expression of FSCN1, which facilitates cytoskeletal remodeling and promotes metastatic behavior of tumor cells. Functional analyses in Chondrosarcoma cell lines and patient-derived samples revealed an inverse correlation between miR-143/145 expression and tumor aggressiveness, further supporting its role as a tumor suppressor [157]. Furthermore, loss of miR-143/145 expression has been linked to the activation of oncogenic signaling pathways, including the PI3K/AKT and MAPK pathways, which contribute to the increased survival, proliferation, and metastasis of Chondrosarcoma cells [157]. The restoration of miR-143/145 expression through mimic therapies has been shown to significantly inhibit the migration, invasion, and EMT of cancer cells, enhancing its therapeutic potential. Furthermore, in in vivo Chondrosarcoma models, the upregulation of miR-143/145 led to a marked reduction in tumor growth and metastatic burden, indicating that this miRNA cluster could serve as both a biomarker and a target for novel therapeutic strategies [157]. Given the resistance of Chondrosarcoma to conventional chemotherapy and radiotherapy, targeting the miR-143/145-FSCN1 axis represents a promising approach to attenuate tumor progression and improve patient outcomes.

Furthermore, **miR-145** has been recognized as a crucial tumor suppressor in Chondrosarcoma, where its downregulation has been demonstrated to enhance tumor cell proliferation, invasion, and metastasis [158]. It has been shown that miR-145 targets SOX9, a major transcription factor essential for chondrogenesis. In Chondrosarcoma, low levels of miR-145 lead to the overexpression of SOX9, which in turn activates ETV5, a transcription factor involved in metastasis, ultimately leading to the increased expression of MMP-2 and increased extracellular matrix degradation [158]. It has been shown that the restoration of miR-145 expression by lentiviral transfection results in the downregulation of SOX9, ETV5, and MMP-2, leading to the reduced invasion and metastasis of Chondrosarcoma cells. These findings establish a miR-145-SOX9-ETV5-MMP-2 regulatory axis, which plays a critical role in tumor invasiveness.

Similarly, **miR-199a** has been revealed as a critical tumor suppressor in Chondrosarcoma, where its downregulation has been demonstrated to contribute to better angiogenesis, metastasis, and tumor cell survival [159]. The chemokine CCL5 has been shown to downregulate the expression of miR-199a, resulting in increased levels of VEGF, a critical mediator of angiogenesis [159]. This CCL5-miR-199a-VEGF axis promotes endothelial cell migration and capillary tube formation, facilitating tumor-associated angiogenesis both in vitro and in vivo. Luciferase reporter assays confirmed that miR-199a directly targets the 3′-UTR of VEGF mRNA, suppressing its translation. Furthermore, in a xenograft model, CCL5 overexpression enhanced vascularization and tumor growth through the downregulation of miR-199a, whereas CCL5 deletion led to increased miR-199a levels, reduced VEGF expression, impaired angiogenesis, and suppressed tumor progression [159].

**miR-34a** has been identified as a key regulator of apoptosis and cellular senescence in Chondrosarcoma, influencing tumor progression through its interaction with Delta-like 1 (DLL1) and the PI3K/AKT signaling pathway [160]. The miR-34a binds directly to the 3′-UTR of DLL1 mRNA, leading to its downregulation. Since DLL1 is a critical ligand of the Notch signaling pathway, its suppression by miR-34a results in reduced levels of PI3K and phosphorylated AKT (p-AKT), thereby impairing cell survival [160]. The overexpression of miR-34a was found to increase caspase-3 and Bax expression, triggering apoptosis, while its inhibition restores DLL1 levels, restores PI3K/AKT signaling and reduces apoptosis. In in vivo models, miR-34a antagonists attenuated chondrocyte apoptosis and cartilage loss, further confirming its role in tumor and cartilage degeneration [160].

**miR-126** has been identified as a tumor suppressor and plays a crucial role in the regulation of cell migration and invasion in Chondrosarcoma [161]. The study showed that the flavonoid Naringin significantly increases the expression of miR-126, leading to the downregulation of VCAM-1 [161]. Results showed that Naringin treatment reduced the motility and invasion ability of Chondrosarcoma cells, effects that were reversed by miR-126 inhibition. Further analysis confirmed that miR-126 directly regulates VCAM-1 expression, suppressing the adhesion and metastatic potential of Chondrosarcoma cells. These results suggest that miR-126 functions as a tumor suppressor by inhibiting VCAM-1-mediated migration and invasion, making it a potential therapeutic target [161]. In addition, the study highlights the potential use of Naringin as an anti-migration agent to counteract the metastatic spread of Chondrosarcoma by modulating miR-126 expression.

In Chondrosarcoma, **miR-631** works as a tumor suppressor [162,163]. Its downregulation has been observed to enhance tumor development, angiogenesis, and metastasis. Bioinformatics and RNA-Seq analysis revealed that miR-631 directly targets Apelin (APLN), a secreted peptide involved in angiogenesis and tumor progression [163]. APLN was found to be highly upregulated in Dox-resistant Chondrosarcoma cells, contributing to their survival and metastatic potential. Functional assays showed that the overexpression of miR-631 led to downregulation of APLN, thereby restoring sensitivity to Dox and increasing apoptosis in resistant Chondrosarcoma cells [163]. In addition, the miR-631-mediated suppression of APLN reduced cancer cell migration and invasion, confirming its role in limiting metastasis.

Recent studies have identified **miR-342-5p** and **miR-491-5p** as tumor suppressors in Chondrosarcoma, demonstrating their ability to induce apoptosis and impair tumor progression [164,165]. It was revealed that both miRNAs directly target anti-apoptotic proteins of the BCL-2 family: miR-342-5p inhibits BCL-2 and Bcl-xL, while miR-491-5p downregulates Bcl-xL and EGFR. Restoring the expression of miR-342-5p and miR-491-5p in Chondrosarcoma cell lines resulted in the increased apoptosis, decreased cell viability, and reduced motility of tumor cells, demonstrating their potent antitumor effects [164]. Notably, miR-342-5p also induced autophagy, further contributing to tumor suppression. In a 3D organoid model of Chondrosarcoma, which mimics the tumor microenvironment in vivo, miR-342-5p uniquely induced tumor cell death, highlighting its potential as a therapeutic target [165].

**Figure 18:**
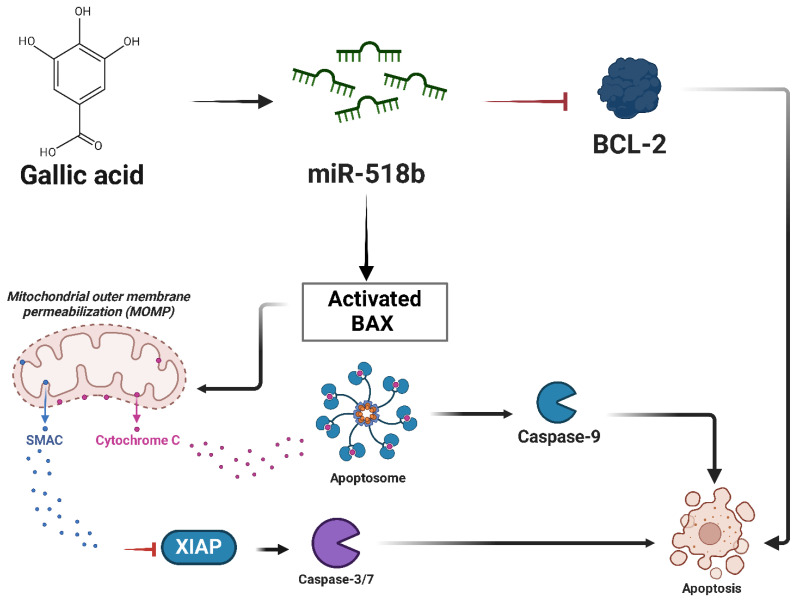
Gallic acid treatment in in vitro models of human Chondrosarcoma induces the increased expression of miR-518b, leading to BCL-2 downregulation and BAX upregulation. This modulation promotes the activation of caspases 3, 7, and 9, key effectors of the apoptotic pathway, ultimately enhancing apoptosis in treated cells.

In Chondrosarcoma, **miR-518b** operates as a tumor suppressor regulating apoptosis and cell migration. A study investigating the effects of gallic acid (GA), a naturally occurring polyphenolic compound, showed that treatment with GA increases the expression of miR-518b in SW1353 human Chondrosarcoma cells, resulting in increased apoptosis and reduced migratory potential [166]. miR-518b was found to modulate mitochondrial apoptosis pathways, as evidenced by the downregulation of BCL-2, upregulation of Bax, and activation of caspase-3 and caspase-9 in GA-treated cells (Figure 18) [166]. It was observed that the restoration of miR-518b in Chondrosarcoma cells significantly inhibited motility and invasion of tumor cells, supporting its role in suppressing metastatic potential. The study suggests that upregulation of miR-518b through natural compounds such as GA could offer a new therapeutic strategy to limit the progression of Chondrosarcoma [166].

## 3. Conclusions

**Figure 19:**
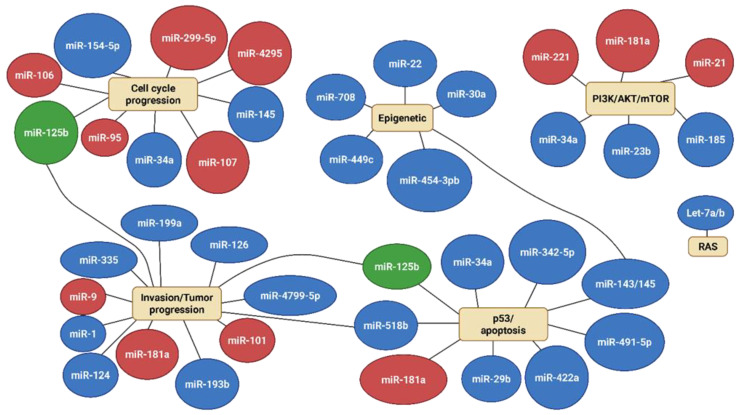
Summary of miRNAs involved in sarcoma-related molecular pathways. The diagram illustrates the major oncogenic and tumor-suppressive miRNAs associated with three main types of bone and soft tissue sarcomas, grouped according to the molecular pathways they regulate. Key signaling pathways include PI3K/AKT/mTOR, p53-mediated apoptosis, cell cycle regulation (E2F/CDK), invasion and tumor progression, and epigenetic/differentiation-related processes. miRNAs are color-coded based on their functional role: red for oncogenic, blue for tumor suppressor, and green for dual-function miRNAs (those reported with both oncogenic and tumor-suppressive activity depending on context). This visual summary highlights the convergence of diverse miRNAs on shared oncogenic pathways, emphasizing their potential as therapeutic targets or biomarkers in sarcoma biology.

A comprehensive understanding of miRNA dysregulation in bone sarcomas underscores their essential role in orchestrating critical cellular processes such as proliferation, apoptosis, differentiation, migration, and metastasis. The miRNAs function as both oncogenes and tumor suppressors, and their expression profiles vary significantly among different subtypes of bone sarcomas, including Osteosarcoma, Ewing’s sarcoma, and Chondrosarcoma. To summarize the roles and regulatory patterns of the various miRNAs analyzed in this review, we constructed an integrated schematic (Figure 19) illustrating the major pathways influenced by both oncogenic and tumor-suppressive miRNAs across bone sarcomas. This visual representation supports the identification of common regulatory nodes and potential miRNA interactions that may influence therapeutic targeting strategies.

This diversity reflects the molecular complexity of these malignancies and underscores the importance of miRNA-mediated regulatory networks in their pathogenesis. From a therapeutic perspective, targeting miRNAs represents a promising frontier in the treatment of sarcomas: strategies such as using miRNA mimics to restore tumor-suppressive miRNAs or using antagomiRs to inhibit oncogenic miRNAs have demonstrated the ability to suppress tumor growth, reduce metastasis, and improve chemosensitivity. For example, the restoration of miR-34a in Osteosarcoma or miR-199a-3p in Ewing’s sarcoma has shown encouraging preclinical results. In recent years, increasing attention has been given to the role of miRNAs in modulating the tumor immune microenvironment (TIME), which is also an emerging area of relevance in bone sarcomas [167,168]. Several miRNAs have been implicated in regulating immune checkpoint molecules, such as PD-L1, as well as influencing cytokine production and immune cell infiltration [169]. These findings suggest that miRNAs could not only serve as biomarkers of immune activity but also act as potential modulators of immunotherapy response. Further research in this direction may open up new therapeutic avenues in sarcoma treatment.

In conclusion, miRNAs have the potential to be a revolutionary pathway in the management of sarcoma, functioning as biomarkers and therapeutic targets. Advancing our understanding of miRNA biology, defining effective delivery mechanisms, and introducing miRNA-based diagnosis and treatment into clinical practice have the potential to change sarcoma care. The resolution of these difficulties will enable the development of more effective and personalized treatments, which will eventually increase outcomes for patients.

## Figures and Tables

**Table 1:** Incidence rates of primary malignant bone sarcomas. Osteosarcoma is the most common subtype, followed by Chondrosarcoma and Ewing’s sarcoma. The incidence percentages are approximate and may vary depending on the population studied and the data source.

Type of Bone Sarcoma	Incidence Percentage (%)
Osteosarcoma	~35–50%
Chondrosarcoma	~20–25%
Ewing sarcoma	~10–15%
Chordoma	~3–5%
Fibrosarcoma of Bone	~2–5%
Adamantinoma	<1%

**Table 2:** List of miRNAs involved in bone sarcoma, highlighting their dual role in oncogenesis and tumor suppression. Some miRNAs function as oncogenes or tumor suppressors depending on the specific bone sarcoma subtype, underscoring their context-dependent regulatory effects.

miRNA	Role	Tumor
**miR-9**	Oncogene	Osteosarcoma
**miR-95**	Oncogene	Osteosarcoma
**miR-106b**	Oncogene	Osteosarcoma
**miR-221**	Oncogene	Osteosarcoma
**miR-299-5p**	Oncogene	Osteosarcoma
**miR-4295**	Oncogene	Osteosarcoma
**miR-17-92**	Oncogene	Osteosarcoma
		
**miR-30a-5p**	Oncogene	Osteosarcoma
**miR-181a**	Oncogene	Osteosarcoma
		Ewing’s sarcoma
		Chondrosarcoma
**miR-21**	Oncogene	Ewing’s sarcoma
**miR-30a**	Oncogene	Ewing’s sarcoma
	Tumor suppressor	Chondrosarcoma
**miR-17-92**	Oncogene	Ewing’s sarcoma
**miR-125b**	Oncogene	Ewing’s sarcoma
	Tumor suppressor	Osteosarcoma
	Tumor suppressor	Chondrosarcoma
**miR-101**	Oncogene	Chondrosarcoma
**miR-34a**	Tumor suppressor	Osteosarcoma
		Ewing’s sarcoma
		Chondrosarcoma
**miR-335**	Tumor suppressor	Osteosarcoma
**miR-199a**	Tumor suppressor	Osteosarcoma
		Chondrosarcoma
**miR-126**	Tumor suppressor	Osteosarcoma
		Chondrosarcoma
**miR-1**	Tumor suppressor	Osteosarcoma
**miR-143**	Tumor suppressor	Osteosarcoma
		Chondrosarcoma
**miR-145**	Tumor suppressor	Osteosarcoma
		Ewing’s sarcoma
		Chondrosarcoma
**miR-422a**	Tumor suppressor	Osteosarcoma
**miR-449c**	Tumor suppressor	Osteosarcoma
		
		Chondrosarcoma
**miR-29b**	Tumor suppressor	Osteosarcoma
**miR-154-5p**	Tumor suppressor	Osteosarcoma
**miR-22**	Tumor suppressor	Ewing’s sarcoma
**miR-145**	Tumor suppressor	Ewing’s sarcoma
**miR-193b**	Tumor suppressor	Ewing’s sarcoma
**miR-185**	Tumor suppressor	Ewing’s sarcoma
**miR-138**	Tumor suppressor	Ewing’s sarcoma
**Let-7a/7b**	Tumor suppressor	Ewing’s sarcoma
**miR-107**	Tumor suppressor	Ewing’s sarcoma
**miR-708**	Tumor suppressor	Ewing’s sarcoma
**miR-199-3p**	Tumor suppressor	Ewing’s sarcoma
**miR-4799-5p**	Tumor suppressor	Chondrosarcoma
**miR-23b**	Tumor suppressor	Chondrosarcoma
**miR-454-3b**	Tumor suppressor	Chondrosarcoma
**miR-342-5p**	Tumor suppressor	Chondrosarcoma
**miR-491-5p**	Tumor suppressor	Chondrosarcoma
**miR-518b**	Tumor suppressor	Chondrosarcoma
**miR-631**	Tumor suppressor	Chondrosarcoma

## Data Availability

All data relevant to the study are included in the article.
